# A retrospective comparative study of local anesthesia only and local anesthesia with sedation for percutaneous endoscopic lumbar discectomy

**DOI:** 10.1038/s41598-022-11393-4

**Published:** 2022-05-06

**Authors:** Liu Yang, Yu-Lin Pan, Chun-Zhi Liu, De-Xin Guo, Xin Zhao

**Affiliations:** 1 Department of Spinal Surgery, Zhengzhou Orthopaedic Hospital, Zhengzhou, Henan Province China; 2 Department of Orthopedics, The Second Hospital of Jilin University, Changchun, Jilin Province China

**Keywords:** Diseases, Medical research, Neurology

## Abstract

It is still an unsolved problem to achieve both immediate intraoperative feedback and satisfactory surgical experience in percutaneous endoscopic lumbar discectomy under local anesthesia for lumbar disk herniation (LDH) patients. Herein, we compared the analgesic and sedative effects of local anesthesia alone and local anesthesia with conscious sedation in LDH patients during percutaneous endoscopic lumbar discectomy. Ninety-two LDH patients were enrolled and divided into the following groups: control group (Con Group), dexmedetomidine group (Dex Group), oxycodone group (Oxy Group), and dexmedetomidine + oxycodone group (Dex + Oxy Group). Various signs, including mean arterial pressure (MAP), heart rate (HR), pulse oximeter oxygen saturation (SpO_2_) and Ramsay score, were compared before anesthesia (T1), working cannula establishment (T2), nucleus pulposus removal (T3), and immediately postoperation (T4). Clinical outcomes, including VAS score, operation time, hospitalization period, Macnab criteria, and SF-36 score, were also evaluated. The Dex + Oxy Group showed the most stable MAP and HR at T2 and T3 in all groups. The clinical outcomes, such as VAS, hospitalization period, Macnab criteria, and SF-36 score, have no significant differences among groups (*p* > 0.05). Local anesthesia combined with conscious sedation is a safe and effective method to improve the surgical experience and achieve satisfying clinical outcomes for LDH patients during percutaneous endoscopic lumbar discectomy.

## Introduction

Lumbar disk herniation (LDH) is a common disease in spinal surgery that often requires surgical treatment^1,2^. Lumbar discectomy can improve pain, function, and quality of life for LDH patients^3^. Conventional open lumbar surgery has been perceived as an effective intervention but carries several disadvantages, including postoperative back pain and a long recovery period. Subsequently, percutaneous endoscopic lumbar discectomy has been developed to facilitate lumbar discectomy^4–6^, with the following advantages such as paravertebral soft tissue protection, shorter hospital stays, less blood loss, and faster patient recovery^6–8^. Consequently, the percutaneous endoscopic technique received increasing attention from spinal surgeons around the worldwide^9^.

Local and general anesthesia are common analgesic methods used in percutaneous endoscopic lumbar discectomy^10–13^. General anesthesia can achieve an excellent surgical experience for patients^11–13^. However, it lacks intraoperative feedback during the operation, which indicates that the unconscious patients cannot communicate with the surgeon if their nerve root or spinal cord was damaged. Therefore, general anesthesia may increase the surgical risk of percutaneous endoscopic lumbar discectomy. Moreover, local anesthesia has the advantage of intraoperative feedback to improve safety during spinal surgery^10^. Nevertheless, it provides a poor surgical experience for pain-sensitive patients, which may cause anxiety and psychentonia, leading to an increase of blood pressure and heart rate, and even cardiovascular or cerebrovascular accidents^10^. Thus, achieving effective pain relief, immediate feedback during operation, and satisfactory surgical experience simultaneously in percutaneous endoscopic lumbar discectomy for patients with LDH remains to be a challenge.

Dexmedetomidine is a new alpha 2 adrenal receptor agonist with good sedative and analgesic effects, which has no inhibiting respiration and is easy to wake up after surgery^14–18^. Gadjradj et al.^19^ has reported that percutaneous transforaminal endoscopic discectomy performed under local anesthesia and conscious sedation using dexmedetomidine is safe and effective in treating sciatica and yields high satisfaction rates from surgeons, anesthesiologists, and patients. Moreover, oxycodone hydrochloride, as the only opioid and double receptor agonist, has an excellent inhibitory effect on mixed somatic and visceral pain^20,21^. We hypothesized that percutaneous endoscopic lumbar discectomy under local anesthesia and conscious sedation, which uses dexmedetomidine combined with oxycodone hydrochloride, could achieve a good analgesic effect and improve the surgical experience intraoperatively. Herein, we evaluated local anesthesia only versus local anesthesia combined with conscious sedation for LDH patients who underwent percutaneous endoscopic lumbar discectomy.

## Results

### Patient characteristics

The demographic characteristics of all 92 patients were compared by chi-square test (gender and the segment of LDH) and one-way ANOVA (age), and the results showed that there was no statistical difference in the demographic characteristics among the four groups.

### Vital signs and sedation

**Figure 1:**
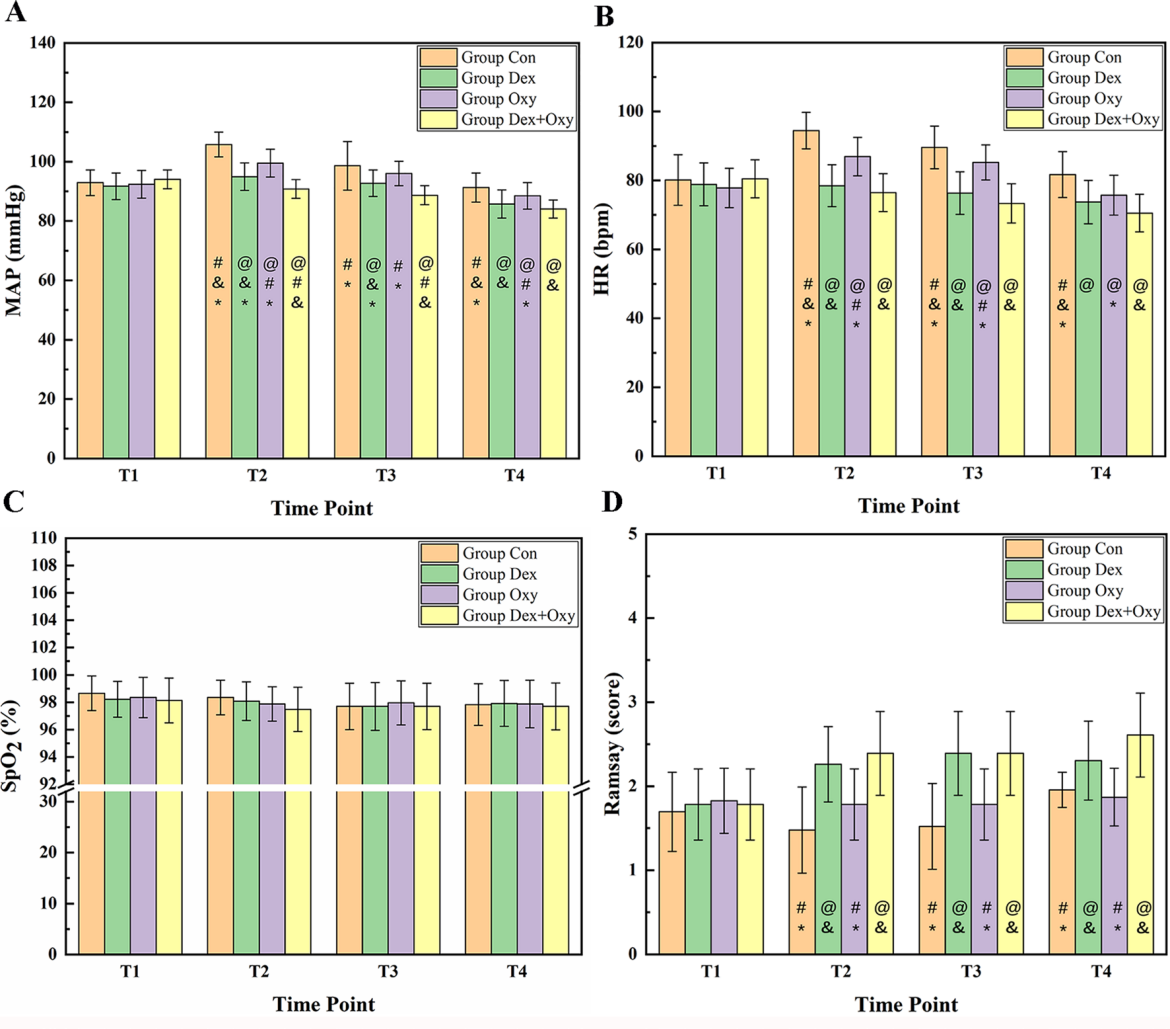
The results of MAP (**A**), HR (**B**), SpO2 (**C**), and Ramsay score (**D**) at T1-T4 in Con Group, Dex Group, Oxy Group, and Dex + Oxy Group. "@", "#", "&", "*" represent the difference was statistical when compared with Con Group, Dex Group, Oxy Group, and Dex + Oxy Group, respectively.

The results of MAP, HR, SpO_2,_ and Ramsay scores at T1, T2, T3, and T4 in four groups are shown in Fig. 1A–D. Firstly, a homogeneity test of variance was done to determine whether one-way ANOVA or Welch ANOVA would be used to compare the values of MAP, HR, SpO2, and Ramsay scores at different time points, and the results are shown in Table 1.

**Table 1:** *P* values of ANOVA for MAP, HR, SpO2, and Ramsay at four time points.

Time point	Items				
	MAP	HR	SpO_2_	Ramsay	
				One-way ANOVA	Welch ANOVA
T1	0.301	0.457	0.623	0.769	–
T2	< 0.001*	< 0.001*	0.196	–	< 0.001*
T3	< 0.001*	< 0.001*	0.937	–	< 0.001*
T4	< 0.001*	< 0.001*	0.974	–	< 0.001*

*MAP* mean arterial pressure, *HR* heart rate, *pulse SpO*_*2*_ oximeter oxygen saturation, *T1* before anesthesia, *T2* working cannula establishment, *T3* nucleus pulposus removal, *T4* immediately postoperation;

*Difference was statistically significant.

At the time point of T1,there was no significant difference in MAP, HR, SpO_2_, and Ramsay scores among the four groups (*p* > 0.05). Results also showed that there was no statistical difference in SpO_2_ among groups at all the time points (*p* > 0.05). There were statistical differences in MAP, HR, and Ramsay in time points of T2, T3, and T4. Thus, LSD was used to compare MAP and HR among groups in time points of T2, T3, and T4 to clarify the differences between groups, and the Games-Howell test was used to compare Ramsay scores among T2, T3, and T4 time points. The results are shown in Table 2.

**Table 2:** The *P* values of LSD and Games-Howell test for MAP, HR, and Ramsay at the time points of T2-T4.

Items group	MAP			HR			Ramsay		
	Group Dex	Group Oxy	Group Dex + Oxy	Group Dex	Group Oxy	Group Dex + Oxy	Group Dex	Group Oxy	Group Dex + Oxy
**T2**									
Group Con	< 0.001*	< 0.001*	< 0.001*	< 0.001*	< 0.001*	< 0.001*	< 0.001*	0.139	< 0.001*
Group Dex	–	< 0.001*	0.001*	–	< 0.001*	0.23	–	0.003*	0.788
Group Oxy	< 0.001*	–	< 0.001*	< 0.001*	–	< 0.001*	0.003*	–	< 0.001*
Group Dex + Oxy	0.001*	P < 0.001*	–	0.23	< 0.001*	–	0.788	< 0.001*	–
**T3**									
Group Con	< 0.001*	0.103	< 0.001*	< 0.001*	0.013*	< 0.001*	< 0.001*	0.248	< 0.001*
Group Dex	–	0.042*	0.012*	–	< 0.001*	0.083	–	< 0.001*	1.000
Group Oxy	0.042*	–	< 0.001*	< 0.001*	–	< 0.001*	< 0.001*	–	< 0.001*
Group Dex + Oxy	0.012*	< 0.001*	–	0.083	< 0.001*	–	1.000	< 0.001*	–
**T4**									
Group Con	< 0.001*	0.033*	< 0.001*	< 0.001*	0.001*	< 0.001*	0.015*	0.730	< 0.001*
Group Dex	–	0.036*	0.190	–	0.266	0.075	–	0.005*	0.160
Group Oxy	0.036*	–	0.001*	0.266	–	0.004*	0.005*	–	< 0.001*
Group Dex + Oxy	0.190	< 0.001*	–	0.075	0.004*	–	0.160	< 0.001*	–

*MAP* mean arterial pressure, *HR* heart rate, *T2* working cannula establishment, *T3* nucleus pulposus removal, *T4* immediately postoperation.

*Difference was statistically significant; “–” The same group;

According to Fig. 1A,B and Table 2, the MAP and HR values at the time point of T2, T3, and T4 were decerased in Oxy Group, Dex Group, and Dex + Oxy Group than those in Con Group, especially in Dex + Oxy Group. According to Fig. 1D and Table 2, Ramsay scores in Dex Group and Dex + Oxy Group were significantly decreased than those in Con Group and Oxy Group at the time point of T2, T3, and T4 (*p* < 0.05).

### Clinical outcomes

#### Operation time and hospitalization period

**Figure 2:**
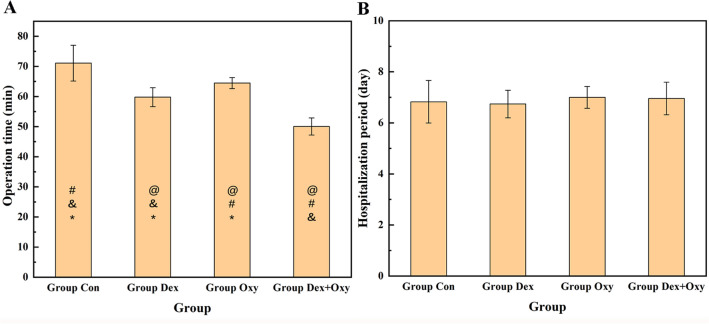
The results of operation time (**A**) and hospitalization period (**B**) in different groups. "@", "#", "&", "*" represent the difference was statistical when compared with Group Con, Group Dex, Group Oxy, and Group Dex + Oxy, respectively.

The results of the homogeneity test of variance in operation time and hospitalization period were *p* > 0.05, and the results of one-way ANOVA showed that there were differences in operation time (*p* < 0.001) and no statistical differences in hospitalization period among different groups (*p* > 0.05). LSD was used to compare the operation time between groups to clarify the difference in operation time between groups (Fig. 2A), and the results showed that the Con Group had the longest operation time, followed by Oxy Group, Dex Group, and Dex + Oxy Group, with significant statistically differences (*p* < 0.001). In the control group, one patient suffered from intense pain accompanied by nervousness, which led to a significant prolongation of the operation time. The results of the hospitalization period are shown in Fig. 2B.

#### VAS score

**Figure 3:**
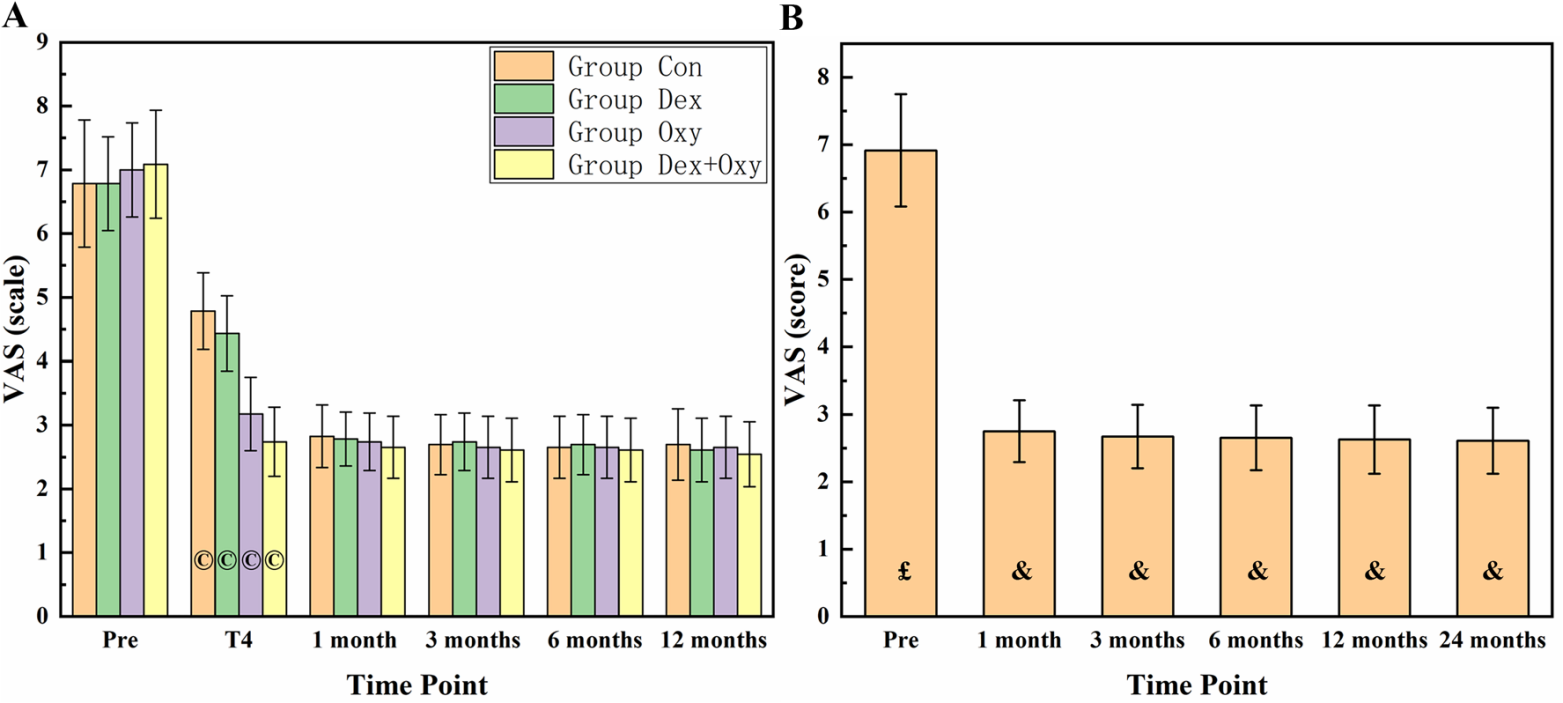
The results of the VAS score at different times of the four groups (**A**). VAS results in follow-up duration (**B**). "©" represents the differences were all statistical when compared with the other groups. "£" represents differences were all statistical when compared with the other time points. "&" represents differences was statistical only when compared with the time point of preoperation.

The VAS scores of the patients in the four groups were evaluated preoperatively and 1 day, 1 month, 3 months, 6 months, 12 months, and 24 months after surgery (Fig. 3A). There was no loss of follow-up at 6 months postoperatively; total 5 patients lost at 24 months follow-up. The results of the homogeneity test of variance were *p* > 0.05. The one-way ANOVA results showed that at the other time points, there was no statistical difference among the 4 groups (*p* > 0.05) (Fig. 3A). Therefore, we combined the data of each group at the same time point and compared the differences in VAS results between different time points to evaluate the therapeutic effect. The result of the homogeneity test of variance was *p* < 0.05, and the result of Welch ANOVA was also *p* < 0.05, so the Games-Howell test was used to compare the difference between every two different time points. The results were shown in Table 3 and Fig. 3B, and significant statistical differences existed between every two time points (p < 0.001), but no significant statistical differences were found between 12 and 24 months postoperatively (*p* > 0.05).

**Table 3:** The *P* values of VAS score between every two time points by LSD.

Time point	preoperation	1 mon- post-op	3 mon- post-op	6 mon- post-op	12 mon- post-op	24 mon- post-op
1 mon- post-op	< 0.001*	–	< 0.001*	< 0.001*	< 0.001*	< 0.001*
3 mon- post-op	< 0.001*	< 0.001*	–	< 0.001*	< 0.001*	< 0.001*
6 mon- post-op	< 0.001*	< 0.001*	< 0.001*	–	< 0.014*	< 0.001*
12 mon- post-op	< 0.001*	< 0.001*	< 0.001*	0.014*	–	0.579
24 mon- post-op	< 0.001*	< 0.001*	< 0.001*	< 0.001*	0.579	–

*VAS* visual analogue scale, *mon-* month or months, *post-op* postoperation.

*Difference was statistically significant; “–” The same time point;

#### SF-36 (PCS + MCS) score, ODI score, and Macnab criteria

**Figure 4:**
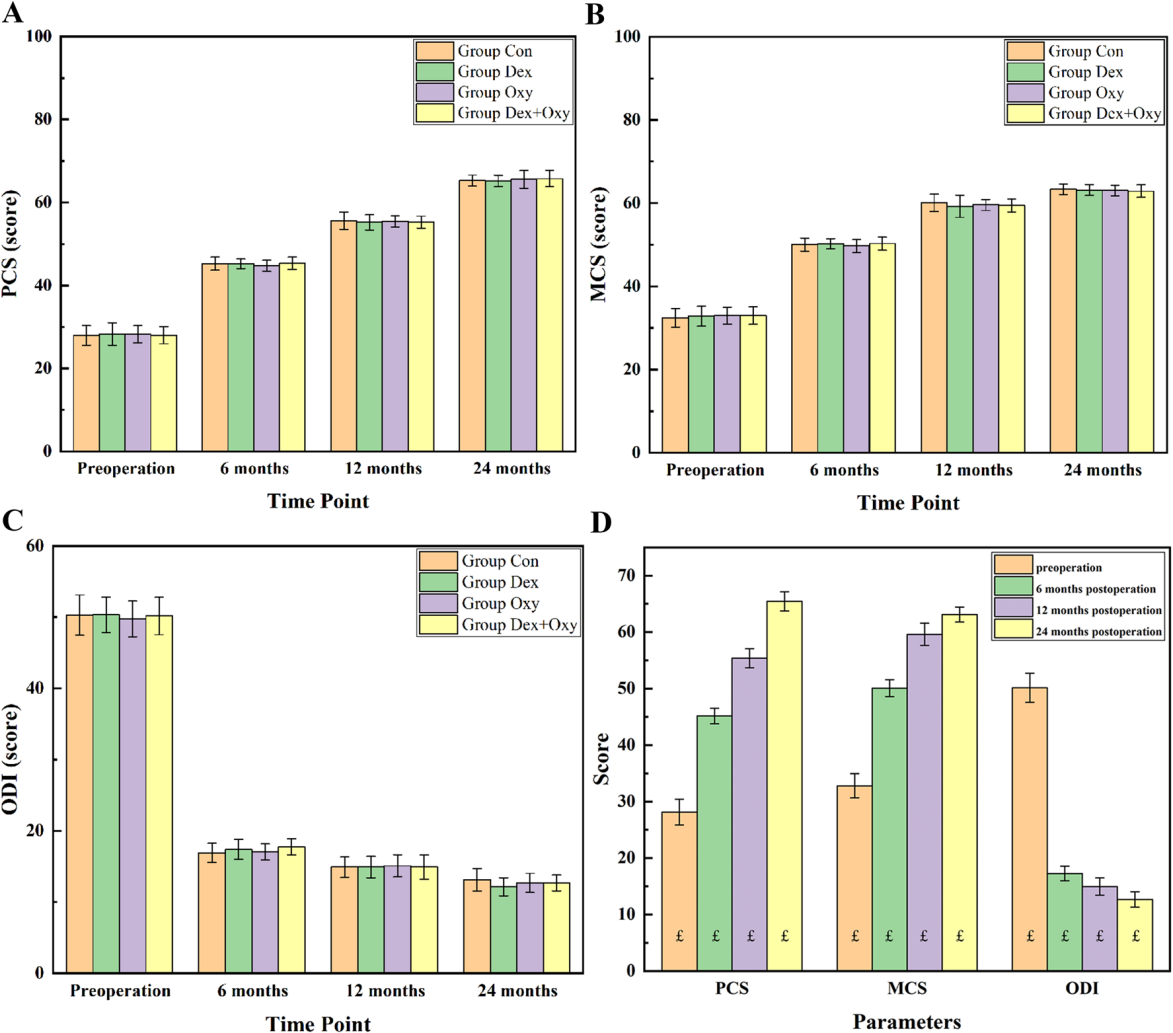
The clinical outcomes of PCS (**A**), MCS (**B**), and ODI (**C**) were shown in different groups. "£" represents differences were all statistical when compared with the other time points.

The SF-36 Physical Component Summary (PCS), the SF-36 Mental Component Summary (MCS), and the ODI score in the four groups were shown in Fig. 4A–C. The results of variance homogeneity and one-way ANOVA results showed no statistical difference among the 4 groups at each same time point (*p* > 0.05). Therefore, we combined the data of the four groups at each same time point as a whole, then compared SF-36 and ODI between different time points to evaluate the clinical outcomes. The results were showed in Fig. 4D.

The clinical outcomes were rated according to modified Macnab criteria as "Excellent" (patient symptom-free, able to resume normal life and work), "Good" (slight symptoms remain, activity slightly limited, no effect on life and work), "Fair" (some symptom relief, activity significantly limited, life and work affected) or "Poor" (post-operation symptoms the same or worse as preoperation symptoms)^22^. The results of modified Macnab criteria at 24 months postoperation were shown in Table 4, Chi-square test was used for comparison among groups, and the *p*-value was more than 0.05. This result showed that local anesthesia with sedation and without sedation did not improve the clinical outcomes.

**Table 4:** The results of modified Macnab criteria at 24 months postoperation.

Group	Poor/total	Fair/total	Good/total	Excellent/total	χ^2^	*P*
Group Con	0/22	0/22	4/22	18/22	0.770	0.857
Group Dex	0/22	0/22	3/22	19/22		
Group Oxy	0/21	0/21	3/21	18/21		
Group Dex + Oxy	0/22	0/22	2/22	20/22		

## Discussion

The essential findings in the present study indicated that local anesthesia with conscious sedation is a safe, effective, and reliable method to achieve satisfying pain control during percutaneous endoscopic lumbar discectomy surgery. Another finding was that dexmedetomidine combined with oxycodone under local anesthesia could reduce anxiety, relieve pain, and improve the surgical experience during percutaneous endoscopic surgery for the treatment of LDH. Both local anesthesia and general anesthesia are effective methods for minimally invasive surgery in spinal surgery. Local anesthesia is inferior to general anesthesia in surgical experience for patients. However, local anesthesia may be superior to general anesthesia in information feedback. According to previous studies, simultaneous intraoperative feedback and good surgical experience remain challenging in LDH patients who underwent percutaneous endoscopic lumbar discectomy^23^. We, herein, applied local anesthesia combined with conscious sedation to improve the surgical experience for LDH patinets in percutaneous endoscopic lumbar discectomy.

Opioids^24–27^ and NSAIDs^28–30^ are two commonly used agents for pain management in clinical practice. Opioids are mainly utilized to relieve pain by binding to the opioid receptors in the central and peripheral nervous systems^31^. However, it has several side effects, including nausea, vomiting, dizziness, itching, sedation, respiratory depression, uroschesis, constipation, euphoria, nausea, vomiting, respiratory depression, excessive sedation, and liver dysfunction^32–37^. Meanwhile, NSAIDs are predominantly applied to inhibit the synthesis of prostaglandins and the release of bradykinin in the process of inflammation to relieve pain^38,39^. Nevertheless, NSAIDs could induce severe gastrointestinal reactions^39^. Therefore, choosing agents with low side effects and good analgesic effects durinng percutaneous endoscopic lumbar discectomy surgery is relatively crucial.

Dexmedetomidine is a highly selective alpha 2 receptor agonist characterized by sedation, analgesia, anti-anxiety, easy arousal, and mild respiratory inhibition^14,16,18^. Mantz et al.^40^ found that dexmedetomidine has a good analgesic effect for controlling acute and chronic inflammatory pain, postoperative pain, and chronic pain. In this study, we found that both Dex Group and Dex + Oxy Group have a lower MAP and HR than Con Group and Oxy Group at the time points of T2, T3, and T4 (*p* < 0.05), indicating that dexmedetomidine plays an essential role in controlling blood pressure and HR, which contribute to alleviating stress response and maintaining stability. Our results are consistent with the previous outcomes conducted by Greenberg et al.^14^.

Regarding the analgesic mechanism of dexmedetomidine, it activates alpha 2 receptors in the presynaptic membrane and inhibits neuronal excitation and norepinephrine release by negative feedback, thus stopping the transmission of pain signals^14,16,18^. Dexmedetomidine can also stimulate 2 receptors in the postsynaptic membrane, inhibit sympathetic activity, cause slow heart rate, decrease blood pressure, and produce sedative and anti-anxiety effects^15,16,18^. Moreover, it can directly bind to 2 receptors in the intramedullary system, exerting analgesic and sedative effects. In our opinion, the stimulation of paravertebral soft tissue, spinal cord, and nerve root during operation is the main cause of poor surgical experience. Thus, the pharmacological mechanism of dexmedetomidine might explain why Dex Group and Dex + Oxy Group have a better surgical experience than Con Group and Oxy Group at the time of T2, T3, and T4.

Oxycodone is a semisynthetic tibrazi derivative of opioid alkaloids^14–18,40^, characterized by the easy crossing of the blood–brain barrier, quick onset of action, long half-life, low affinity for receptors, mild respiratory inhibition, mild adverse reactions, little effect on hemodynamics, strong sedative and good analgesic effect^20,21^. In the current study, we found that the Oxy Group has a better analgesic effect than the Con Group, a similar analgesic effect compared to the Dex Group, and a worse analgesic effect than the Dex + Oxy Group. Our results were consistent with a previous study provided by Han et al.^21^, who reported that oxycodone had better analgesic effects, lower incidence of adverse complications, and less analgesic drug consumption than sufentanil in pain management postoperatively. Thus, we believe that oxycodone is an effective agent in suppressing pain but less effective in anti-anxiety. In the present study, we found that the values of MAP and HR in the Oxy Group were lower than those in the Con Group. In our opinion, oxycodone has a good analgesic effect, and this view was supported by authors^20,21^. Besides, the application of oxycodone combined with dexmedetomidine can play a synergistic analgesic effect durting minimal invasive spinal surgery.

We found that MAP and HR in the Con Group were significantly higher than that in other groups during the operation steps of catheter insertion and nucleus removal. We believe that this is due to the pain and anxiety caused by surgical manipulations. In addition, we detected that HR and MAP in the Dex Group and Dex + Oxy Group were significantly lower than that in the Con Group and Oxy Group during catheter insertion and nucleus removal. This result indicates that dexmedetomidine could reduce sympathetic activity, control heart rate and blood pressure, and reduce myocardial oxygen consumption, which is beneficial to alleviating anxiety and improving the surgical experience intraoperatively.

Pain is produced by the coordinated action of the central and peripheral nerve conduction systems^25,41^. Thus, the combined application of multiple analgesic strategies for pain management has been developed^42–45^. Nevertheless, there are numerous side effects of combined analgesic strategies, including nausea, vomiting, pruritus, and even excessive sedation. In our study, the Dex + Oxy Group showed lower side effects and provided a prolonged analgesia effect than the control group. This viewpoint was also consistent with previous studies^46,47^. Moreover, the blood oxygen saturation in the Dex + Oxy Group had no significant defference between intraoperative and preoperative (*p* > 0.05), demonstrating that the application of dexmedetomidine combined with oxycodone did not increase the risk of intraoperative respiratory depression and oxygen saturation reduction.

The Dex Group, Oxy Group, and Dex + Oxy Group had a shorter operative time than the Con Group (*p* < 0.05). We believe that the result is directly related to the patient's cooperation with the surgeon intraoperatively. However, the ODI score, Macnab score, and SF-36 score showed no significant differences among groups during the follow-up visits (*p* > 0.05). Thus, we believe that the local anesthetic combined with conscious sedative in percutaneous endoscopic lumbar discectomy for LDH cannot facilitate the surgical effect but might improve the patient's surgical experience.

In the control group, a patient was very nervous, anxious, and even shouting intraoperatively, which seriously affected the operation, resulting in a prolonged operation time. Unfortunately, bleeding causes blurred endoscopic vision and eventually leads to a dural tear in this patient. Bradycardia occurred in two patients after sedation with dexmedetomidine, and atropine (0.5 mg) was given for symptomatic treatment, then the HR returned to a normal level. No other complications, such as vascular injury, nerve injury, visceral injury, infection, nonunion of incisions, or relapsed LDH, were found.

Positive clinical results were achieved in this study. However, several limitations still deserve our attention. In the present study, there was no pharmacodynamics and pharmacokinetics evaluation of the combined therapy. Thus, pharmacodynamics and pharmacokinetics evaluation for the combination of oxycodone and dexmedetomidine in percutaneous endoscopic lumbar discectomy could be further studied.

Local anesthesia combined with conscious sedation is a safe and effective method to improve the surgical experience and achieve satisfying clinical outcomes for LDH patients who underwent percutaneous endoscopic lumbar discectomy.

## Methods

### Subjects and groups

This is a multicenter, retrospective study conducted at Zhengzhou orthopaedic hospital, Zhengzhou, Henan Province, China; and the second hospital of Jilin University, Changchun, Jilin Province, China. From January 2016 to June 2019, a total of 92 consecutive single-level LDH patients underwent percutaneous endoscopic lumbar discectomy under local anesthesia only or local anesthesia with conscious sedation. Patients were divided into four groups, including Con Group, Dex Group, Oxy Group, and Dex + Oxy Group.

All four groups were given local infiltration anesthesia with 1% lidocaine 30 ~ 40 mL layer by layer on the skin, subcutaneous fascia, muscle, and articular process. In the Dex + Oxy Group, oxycodone hydrochloride (1 mL/10 mg) was slowly injected intravenously (0.05 mg/kg) (Duration: 1 min) at 10 min before operation; Dexmetomidine hydrochloride (2 mL/200 μg) (0.5 μg/kg) was infused continuously for 10 min, and then maintained at a rate of 0.4 μg/ kg·h until the end of the operation. In the Dex Group, 0.3 mL of normal saline was slowly injected at 10 min before operation; Dexmetomidine hydrochloride (2 mL/200 μg) (0.5 μg/kg) was infused continuously for 10 min and then maintained at a rate of 0.4 μg/kg/h until the end of the operation. In the Oxy Group, oxycodone hydrochloride (1 mL/10 mg) was slowly injected intravenously (0.05 mg/kg) (Duration: 1 min) at 10 min before operation; 0.3 mL of normal saline was infused continuously for 10 min, and then maintained at a rate of 0.4 μg/kg·h until the end of the operation. In the Con Group, 0.3 mL of normal saline was slowly injected intravenously at 10 min before the operation; 0.3 mL of normal saline was infused continuously for 10 min and then maintained at a rate of 0.4 μg/kg h until the end of the operation. Subsequently, all patients underwent percutaneous endoscopic lumbar discectomy via the foraminal approach^48^. During the operation, if the heart rate was less than 50 beats/min, atropine (0.5 mg) would be given for symptomatic treatment.

### Evaluation parameters

Vital signs, including MAP, HR, and SpO_2_, were compared at T1, T2, T3, and T4. The Ramsay score is a 6-level clinical score that scores the patients’ level of sedation on a scale from 1 (patient is anxious and agitated or restless or both) to 6 (patient exhibits no response to stimulus)^49^. Moreover, the clinical outcomes, including the hospitalization period, VAS score, the SF-36 score, ODI score, and Macnab criteria, were evaluated. Telephone follow-up was performed at 1 and 3 months after surgery, and patients were followed up at the hospital from 6 to 24 months after surgery. The X-rays, CT, and MRI of the lumbar were performed preoperatively. All of the LDH patients treated with percutaneous endoscopic lumbar discectomy underwent routine X-ray examination in the outpatient department during follow-up visits at 1 month and 6, 12, and 24 months postoperatively.

### Statistical methods

SPSS (version 26.0) was used for statistical analysis. The chi-square test was used for counting data and analysis of variance (ANOVA) for measurement data. Before ANOVA, a homogeneity test of variance was performed. If *p* > 0.05, one-way ANOVA would be performed. If *p* < 0.05, Welch ANOVA would be used for statistical analysis. Least-Significant Difference (LSD) was used to test the differences between groups if the results of one-way ANOVA showed that *p* < 0.05, and the Games-Howell test was used to test the differences between groups if the results of Welch ANOVA showed that *p* < 0.05. *p* < 0.05 is considered to be a statistical difference. For the inter-group comparison of multiple groups, the corrected p* is used for comparison according to the number of pair comparisons (k), p* = *p*/k.

### Ethical approval and consent to partcipate

This study was based on the principles outlined in the Helsinki Declaration, which the Ethics Committee approved of Zhengzhou Orthopaedic Hospital (No.2021014). All volunteers who participated in the study signed written informed consent.

## Data Availability

The datasets used during the current study are available from the corresponding author on reasonable request.

## Abbreviations

LDH: Lumbar disk herniation
Dex: Dexmedetomidine group
Oxy: Oxycodone group
VAS: Visual analogue scale
MAP: Mean arterial pressure
HR: Heart rate
SpO_2_: Pulse oximeter oxygen saturation
SF-36: Short form-36 health survey questionnaire
ODI: Oswestry disability index
CT: Computed tomography
MRI: Magnetic resonance imaging
